# Racial and Ethnic Differences in Out-of-Pocket Spending for Maternity Care

**DOI:** 10.1001/jamahealthforum.2024.5565

**Published:** 2025-02-28

**Authors:** Rebecca A. Gourevitch, Jessica L. Cohen, Tara Shakley, Katie Camacho Orona, Sung Min Park, Mary Beth Landrum, Meredith B. Rosenthal, Mark W. Friedberg, Anna D. Sinaiko

**Affiliations:** 1 Department of Health Policy and Management, University of Maryland, College Park; 2 Department of Global Health and Population, Harvard T. H. Chan School of Public Health, Boston, Massachusetts; 3 Blue Cross Blue Shield of Massachusetts, Boston, Massachusetts; 4 Department of Health Policy and Management, Harvard T. H. Chan School of Public Health, Boston, Massachusetts; 5 Department of Health Care Policy, Harvard Medical School, Boston, Massachusetts

## Abstract

**Importance:**

Rising out-of-pocket costs of maternal health care for people with commercial insurance may affect use of health care and outcomes. There are stark racial and ethnic disparities in outcomes, but little is known about differences in spending.

**Objective:**

To measure differences in out-of-pocket spending for maternity care by race and ethnicity.

**Design, Setting, and Participants:**

This retrospective cross-sectional study used administrative data from Blue Cross Blue Shield of Massachusetts from January 1, 2018, through December 31, 2022, for pregnancies, deliveries, and 42-day postpartum care. Participants were continuously enrolled during pregnancy, delivery, and 42 days post partum (collectively termed *maternity episode*).

**Exposures:**

The primary characteristic of interest was the birthing person’s race and ethnicity.

**Main Outcomes and Measures:**

The primary outcome was total out-of-pocket spending during the maternity episode. Out-of-pocket spending was measured separately for the pregnancy period, prenatal services, and delivery, by type of cost sharing, and as a percentage of the median household income in the patient’s census tract (using American Community Survey data). Race and ethnicity were measured via self-report and imputation.

**Results:**

The analytic sample included 87 253 maternity episodes among 76 826 unique birthing persons (mean [SD] age, 32.4 [4.7] years; 99.8% female) between 2018 and 2022; among maternity episodes, 8572 birthing persons (9.8%) were Asian, 3331 (3.8%) were Black, 6872 (7.9%) were Hispanic, and 68 478 (78.5%) were White. Mean out-of-pocket spending for the maternity episode was highest among Black birthing people ($2398 [$426]), followed by Hispanic ($2300 [$572]), Asian ($2202 [$603]), and White ($2036 [$1547]) birthing people (*P* < .001). These differences remained statistically significant after adjusting for health and demographic characteristics. The differences were largest in the prenatal period and for coinsurance payments. Black (1003 [30.1%]) and Hispanic (2302 [33.5%]) birthing people were more likely than Asian (1569 [18.3%]) and White (12 600 [18.4%]) birthing people to be enrolled in plans with high coinsurance, but not plans with high deductibles (3317 [38.7%] for Asian, 1232 [37.0%] for Black, 2350 [34.2%] for Hispanic, and 24 515 [35.8%] for White birthing people).

**Conclusions and Relevance:**

In this study, differences in out-of-pocket maternity spending among the commercially insured were associated with differences in coinsurance rates. These costs could lead people to forgo needed health care or other basic needs that support health (eg, food or housing). Changes to health plan benefit design could improve equity in out-of-pocket maternity spending and its consequences.

## Key Points

**Question:**

Are there differences in out-of-pocket spending for maternity care across racial and ethnic groups?

**Findings:**

In this cross-sectional study of 76 826 birthing people with commercial insurance, Asian, Black, and Hispanic patients spent more out-of-pocket on maternity care than White patients, partially driven by enrollment in plans with higher coinsurance levels.

**Meaning:**

These results suggest that changes to benefit design, particularly coinsurance levels, could reduce disparities in out-of-pocket spending for maternity care and their consequences.

## Teaser

This cross-sectional study explores differences in out-of-pocket spending for maternity care among birthing people of different racial and ethnic groups.

## Introduction

There are stark differences in maternal health outcomes by race and ethnicity in the US, including severe maternal morbidity, stillbirth, and preterm birth.^1,2,3,4^ Race is a social construct that is associated with exposures to systemic, interpersonal, historical, and/or contemporary racism and policies that affect how people are treated and their social determinants of health.^5^ Black and Hispanic birthing people have lower rates of adequate prenatal care and are more likely to deliver at lower-quality hospitals than White birthing people.^5,6^ Less is known about whether out-of-pocket spending during maternity care differs by race and ethnicity. Differences in spending could be a promising target for intervention because they are directly influenced by insurance benefit design.

Half of childbirths in the US are covered by commercial insurance plans, and most have some cost sharing for maternity care.^7,8^ Although the Affordable Care Act made prenatal visits and some associated preventive services exempt from cost sharing,^9^ important prenatal services (eg, blood tests, ultrasonographic examination, and fetal surveillance for common morbidities) and delivery hospitalizations are not exempt. In 2018 to 2020, birthing people with commercial insurance paid an average of $3070 out-of-pocket during the prenatal and delivery periods,^10^ and higher deductibles have driven increases in these costs.^7^ Out-of-pocket costs can be barriers to use of health care services.^11,12^ Giving birth increases the likelihood of having catastrophic health care expenditures and problems paying medical bills, particularly among patients with pregnancy complications or who are in low-income groups,^13,14,15,16,17,18,19^ and may increase challenges paying for other basic household expenses.

Despite evidence of high and growing out-of-pocket costs and their consequences, little is known about whether out-of-pocket costs for maternity care vary by race and ethnicity. In this report, we describe differences in out-of-pocket spending during maternity episodes by birthing peoples’ race and ethnicity using data from Blue Cross Blue Shield of Massachusetts (BCBSMA) from January 1, 2018, to December 31, 2022. Due to long-standing historical differences in income by race and ethnicity in the US,^20^ we explore differences in out-of-pocket spending both as a total and a percentage of median census-tract household income. We also examine differences in out-of-pocket spending by part of the maternity episode and by type of cost sharing, as well as differences in benefit design across racial and ethnic groups.

## Methods

### Data

This retrospective cross-sectional study uses health plan enrollment and medical claims data from 2018 to 2022 from BCBSMA, a large health plan that covers approximately 3 million individuals per year. We obtained annual median household income by census block group from the US Census American Community Survey for these years. We used data from the natality records in the Centers for Disease Control and Prevention WONDER (Wide-ranging Online Data for Epidemiologic Research) databases to compare the racial and ethnic composition of our sample with commercially insured births in Massachusetts, New England, and nationally. We followed the Strengthening the Reporting of Observational Studies in Epidemiology (STROBE) reporting guideline for cross-sectional studies and received an exempt determination from the institutional review board at the Harvard T.H. Chan School of Public Health, Boston, Massachusetts, for ethical approval and informed consent.

### Key Variables

Data on birthing person race and ethnicity were obtained from 2 sources. First, we used self-reported race and ethnicity that members provided directly to BCBSMA, whenever these were present (19% of members). For all others (81% of members), race and ethnicity were imputed using an expanded version of RAND Corporation’s Bayesian Improved First Name Surname and Geocoding method that incorporated full name and address, individual race and ethnicity reported in the state vaccine registry data, and other variables available to health plans (eg, gender).^21^ The imputation model was trained using a subsample of BCBSMA members who had voluntarily self-reported their race and ethnicity directly to the plan at the time of this study. The performance of the imputation model was assessed using the remaining members who had self-reported their race and ethnicity but were not included in the subsample used to train the algorithm. Compared with self-report, imputed race and ethnicity had moderate to high sensitivity (ranging from 74.8% for Hispanic enrollees to 99.1% for White enrollees) and high specificity for all race and ethnicity categories (81.9% for White enrollees to 99.5% for Black enrollees) (eTable 1 in Supplement 1). BCBSMA uses these race and ethnicity data to measure and reward health systems for improvements in health equity.^22^

Race and ethnicity data were combined into a single variable, per federal guidelines.^23^ Birthing people of Hispanic or Latino ethnicity were grouped as Hispanic regardless of race. The remaining non-Hispanic birthing people were categorized by race as follows: American Indian or Alaska Native, Asian, Black, Native Hawaiian or Other Pacific Islander, White, other race, and multiracial.

### Study Sample

We included all deliveries where the birthing person was enrolled in a BCBSMA commercial health plan for their full maternity episode, defined as the prenatal period, the delivery period, and 42 days post partum (eFigure in Supplement 1). We required continuous enrollment to observe all out-of-pocket spending on care during pregnancy and delivery. The delivery period was identified as hospitalization with a delivery code (eTable 2 in Supplement 1) and measured as date of admission through date of discharge. For the prenatal period, we included the number of weeks of gestation prior to delivery as indicated by the *International Statistical Classification of Diseases, Tenth Revision* (*ICD-10*) gestational age code on the birthing person’s delivery claims. The postpartum period included the 42 days following the date of discharge on the delivery admission to capture out-of-pocket spending due to complications from delivery.^24^

We included deliveries where the birthing person’s race and ethnicity were self-reported as Asian, Black, Hispanic, or White or where the imputed race and ethnicity had the highest probability in 1 of these 4 categories. Only 0.84% of deliveries were categorized as other races, including American Indian or Alaska Native, Native Hawaiian or Other Pacific Islander, other, and multiracial. We excluded these other race categories because the sample sizes per group were too small to produce reliable estimates. We excluded deliveries that occurred in US states with fewer than 50 deliveries during the study period because some models include controls for state of residence.

### Study Outcomes

The primary study outcome was the birthing person’s out-of-pocket spending, measured as the sum of deductible, coinsurance, and copayments for covered medical services. Prescription drug spending was not included because not all enrollees in the sample had prescription drug coverage through BCBSMA. We measured out-of-pocket spending for all care provided during the prenatal period, delivery, 42 days post partum, and total maternity episode. We also measured out-of-pocket spending on a subset of care during the prenatal period that we termed *prenatal services*, which included routine obstetrical radiological examinations and laboratory tests, genetic testing for pregnancy, fetal testing, and care where the servicing clinician was an obstetrician or gynecologist (eTable 3 in Supplement 1). We disaggregated out-of-pocket spending by type of payment (ie, deductible, copayments, or coinsurance). All spending was inflated to 2022 US dollars using the medical consumer price index.^25^ Our secondary outcome was out-of-pocket spending as a percentage of the median household income in the birthing person’s census block group, measured using income data in the year of delivery.

### Covariates

Our analyses included additional covariates that have been shown to impact utilization of and spending on maternity care. We included the birthing person’s categorical age at delivery and their state or Census division of residence during the year of delivery. We included whether a delivery was by cesarean delivery using billing codes (eTable 2 in Supplement 1). We measured clinical risk of each delivery using quartiles of the Leonard Comorbidity Index (ie, quartile 1 indicates lowest risk; quartile 4, highest risk), a validated index that uses *ICD-10* diagnoses codes to capture risk of severe maternal morbidity.^26^ We also measured length of stay during the delivery hospitalization and categorized deliveries by tertile of length of stay within delivery mode (vaginal or cesarean delivery). Finally, we categorized each person’s health plan benefit design by their deductible level ($0, $1-$1000, or >$1000) and inpatient coinsurance level (0%, 1%-10%, or >10%).

### Statistical Analysis

The analysis was conducted at the maternity episode level. We provide descriptive statistics of the study cohort characteristics. We report out-of-pocket spending on prenatal services, during the prenatal period, during delivery, during the postpartum period, and on the total maternity episode, stratified by race and ethnicity of the birthing person. To incorporate uncertainty about imputed race and ethnicity data, we computed weighted means for each outcome: the weights from the imputation model were the vector of probabilities of the birthing person being from each race and ethnicity category (with self-reported race and ethnicity receiving a weight equal to 1). For the outcome of out-of-pocket spending as a percentage of the median household income in the birthing person’s census tract, we report weighted summary statistics (mean, median, IQR, and 10th-90th percentile range) by race and ethnicity.

To estimate whether racial and ethnic differences in out-of-pocket spending during the maternity episode persist after controlling for covariates that impact health care utilization (and, by extension, out-of-pocket spending), we conducted regression analyses using multiple imputation. Specifically, we created 10 imputed datasets by randomly assigning each member to a race and ethnicity category according to the vector of individual-level probabilities that a member was from each group. We then regressed out-of-pocket spending on race and ethnicity, controlling for covariates, in each imputed dataset and combined results using standard formulas.^27^ We used linear regression models because estimated coefficients can be interpreted as differences in mean spending, which we believe is the policy-relevant parameter. With sufficient sample size, linear models provide robust estimates of regression coefficients without the assumption of normality.^28,29^ We conducted 2 sensitivity analyses of this model specification. First, we clustered SEs at the member level to account for some members having multiple maternity episodes in the sample. Second, we used a generalized linear model with a gamma distribution and log-link to examine sensitivity to functional form. All analyses were conducted in SAS, version 9.4 (SAS Institute Inc). Two-sided *P* < .05 indicated statistical significance.

## Results

The study sample included 87 253 deliveries (76 826 unique individuals; mean [SD] age, 32.4 [4.7] years; 87 066 [99.8%] female, 21 [<0.1%] male, 4 [<0.1%] nonbinary, and 162 [0.2%] with missing gender data); among maternity episodes, 8572 birthing persons (9.8%) were Asian, 3331 (3.8%) were Black, 6872 (7.9%) were Hispanic, and 68 478 (78.5%) were White (Table 1). The racial and ethnic composition of our sample was similar to that of commercially insured births in Massachusetts and in New England, with a slightly higher proportion of White births (78.5% in our sample vs 71.2% in Massachusetts and 76.0% in New England) (eTable 4 in Supplement 1). In the sample, 38 378 (44.0%) of deliveries were to birthing people aged 30 to 34 years, 28 188 (32.3%) were by cesarean, 23 029 (26.4%) were in states outside New England, and 23 501 (26.9%) were to birthing people living in census block groups with median household income of $75 000 or less.

**Table 1.** Characteristics of the Study Population

Characteristic	Maternity episodes, No. (%) (N = 87 253)
No. of birthing people^a^	76 826
Race and ethnicity	
Asian	8572 (9.8)
Black	3331 (3.8)
Hispanic	6872 (7.9)
White	68 478 (78.5)
Age group, y	
<20	676 (0.8)
20-24	4896 (5.6)
25-29	14 956 (17.1)
30-34	38 378 (44.0)
35-39	23 543 (27.0)
40-44	4466 (5.1)
>44	338 (0.4)
Gender^b^	
Female	87 066 (99.8)
Male	21 (<0.1)
Nonbinary	4 (<0.1)
Maternity episode clinical characteristics	
Leonard Comorbidity Index, mean (SD)^c^	14 (19.17)
Cesarean delivery	28 188 (32.3)
Median household income in census tract, US $^d^	
≤55 000	9808 (11.2)
55 001-75 000	13 693 (15.7)
75 001-95 000	15 785 (18.1)
>95 000	42 343 (48.5)
Census division	
New England	64 224 (73.6)
Middle Atlantic	4427 (5.1)
East North Central	3257 (3.7)
West North Central	1498 (1.7)
South Atlantic	5447 (6.2)
East South Central	920 (1.1)
West South Central	2439 (2.8)
Mountain	1860 (2.1)
Pacific	3181 (3.6)
Delivery year, No. (%)	
2018	17 576 (20.1)
2019	16 995 (19.5)
2020	17 209 (19.7)
2021	17 869 (20.5)
2022	17 604 (20.2)

^a^ The number of maternity episodes is larger than the number of birthing people because people could have more than 1 maternity episode during the study period.

^b^ Data on gender were missing for 162 individuals (0.2%).

^c^ The Leonard Comorbidity Index is included in the model as quartiles, with 25% of the sample in each quartile. Scores range from 0 to 297, with higher scores indicating greater morbidity.

^d^ Median household income in census tract is missing for 5642 members (6.4%) without address information.

**Figure 1.**
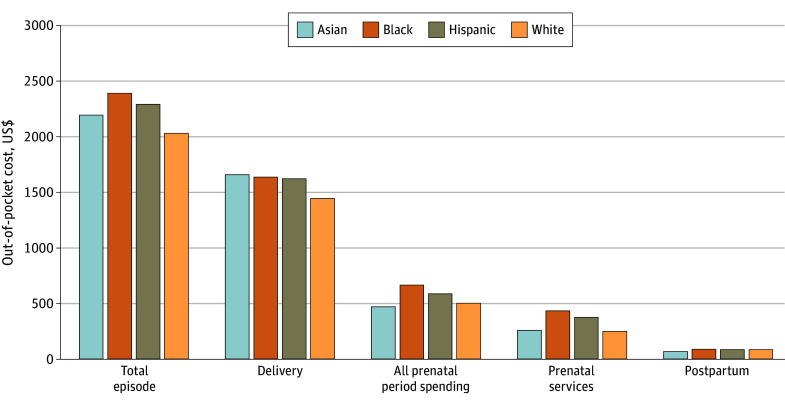
Mean Out-of-Pocket Spending During a Maternity Episode by Race and Ethnicity The total maternity episode includes the prenatal period, delivery, and 42 days post partum (and therefore the delivery and prenatal amounts do not sum to the total). Spending was inflated to 2022 US dollars using the Medical Consumer Price Index. All differences across race and ethnicity groups within spending categories were statistically significant (*P* < .001). See eTable 3 in Supplement 1 for the list of services classified as prenatal services.

Birthing people who were Black or Hispanic had higher mean (SD) out-of-pocket spending over the maternity episode ($2398 [$426] and $2300 [$572], respectively) than birthing people who were Asian ($2202 [$603]), and people who were White had the lowest mean out-of-pocket spending ($2036 [$1547]) (*P* < .001) (Figure 1; eTable 5 in the Supplement 1 presents median spending). These differences correspond to 8.1% more spending for Asian birthing people, 17.7% more for Black birthing people, and 12.9% more for Hispanic birthing people compared with White birthing people. During the prenatal period, Asian birthing people spent 6.3% less (mean [SD], $472 [$259]), Black birthing people spent 32.6% more (mean [SD], $667 [$224]), and Hispanic birthing people spent 16.9% more (mean [SD], $588 [$277]) on all care than did White birthing people (mean [SD], $503 [$691]) (*P* < .001). For recommended prenatal care services, Asian birthing people spent 4.2% more (mean [SD], $259 [$251]), Black birthing people spent 74.4% more (mean [SD], $434 [$247]), and Hispanic birthing people spent 51.2% more (mean [SD], $376 [$310]) than White birthing people (mean [SD], $249 [$675]). Differences in out-of-pocket spending among Black, Hispanic, and White birthing people on delivery and in the postpartum period were smaller. Black and Hispanic birthing people’s out-of-pocket spending on maternity care also represented a higher proportion of median household income in their census tract (4.1% for Black and 3.6% for Hispanic compared with 2.6% for Asian or 2.4% for White birthing people; *P* < .001) (eTable 6 in Supplement 1).

Table 2 compares differences in out-of-pocket spending across race and ethnicity groups with and without adjusting for birthing person’s age, pregnancy health, time, and geography. After adjusting for these factors, race and ethnicity remained associated with out-of-pocket spending for the maternity episode, though differences were smaller. In adjusted analyses, out-of-pocket spending per maternity episode for deliveries among Black birthing people was $251.88 (95% CI, $190.03-$313.72) greater, spending for deliveries among Hispanic birthing people was $91.95 (95% CI, $47.22-$136.68) greater, and spending for deliveries among Asian birthing people was $122.34 (95% CI, $82.33-$162.35) greater than for deliveries among White birthing people (eTable 7 in Supplement 1 presents full regression results; eTables 8 and 9 in Supplement 1 present sensitivity analyses, findings of which were consistent).

**Table 2.** Maternity Episode Out-of-Pocket Spending Differences Between Race and Ethnicity Groups^a^

Race and ethnicity group	Unadjusted		Adjusted		
	Coefficient (95% CI), $	Unadjusted estimated out-of-pocket spending (95% CI), $	Coefficient (95% CI), $	Unadjusted estimated out-of-pocket spending (95% CI), $
Asian	120.58 (107.55-133.61)	2172.88 (2160.60-2185.16)	122.34 (82.33-162.35)	2191.72 (2188.20-2195.25)
Black	360.48 (340.32-380.64)	2412.78 (2393.09-2432.46)	251.88 (190.03-313.72)	2319.62 (2316.09-2323.15)
Hispanic	254.43 (240.04-268.82)	2306.73 (2293.01-2320.44)	91.95 (47.22-136.68)	2160.85 (2157.33-2164.38)
White	0 [Reference]	2052.29 (2047.95-2056.64)	0 [Reference]	2069.09 (2065.57-2072.62)

^a^ Regression analyses used multiple imputation to account for imputed data; as a result, the unadjusted results may not be equal to the raw means reported in Figure 1. Spending inflated to 2022 dollars using the Medical Consumer Price Index. See eTable 7 in Supplement 1 for full regression results. Adjusted results based on linear regression models including controls for age, quartile of Leonard Comorbidity Index score, cesarean delivery, census division, delivery length of stay tertile, and month-year interactions. All differences between each race and ethnicity group and the reference group (White) were statistically significant at *P* < .001.

**Figure 2.**
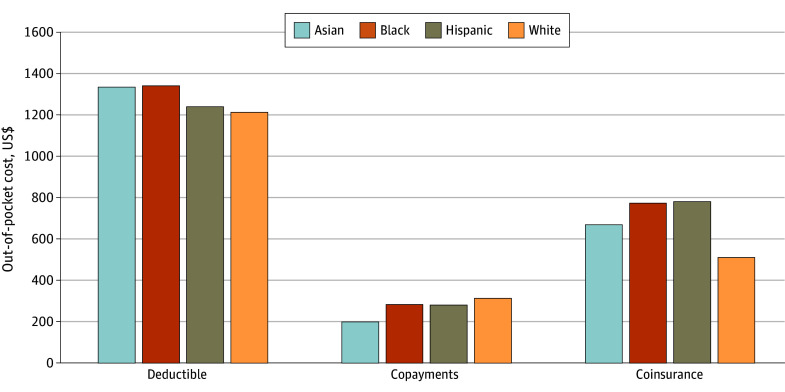
Mean Maternity Episode Out-of-Pocket Spending by Type of Cost Sharing The maternity episode includes the prenatal period, delivery, and 42 days post partum (see eTable 10 in Supplement 1 for prenatal, delivery, and postpartum periods, separately). All differences across race and ethnicity groups within spending categories were statistically significant (*P* < .001). Categories may not sum to the total out-of-pocket spending values (Figure 1) because means were taken within each category of cost sharing. Spending was inflated to 2022 US dollars using the Medical Consumer Price Index.

The biggest differences in out-of-pocket spending across race and ethnicity groups by type of cost sharing were in coinsurance payments. Mean (SD) coinsurance payments for maternity episodes among Asian ($669 [$330]), Black ($772 [$239]), and Hispanic ($779 [$340]) birthing people were higher than among White birthing people ($511 [$814]) (*P* < .001) (Figure 2; eTable 10 in Supplement 1 presents results by prenatal and delivery episode). Levels of deductible payments were lower for Hispanic (mean [SD], $1238 [$441]) and White (mean [SD], $1210 [$1237]) patients (*P* < .001). Levels of copayments were similar across groups and lowest among Asian birthing people (mean [SD], $199 [$108]) (*P* < .001). Similarly, the largest differences in plan benefit design by race and ethnicity were in coinsurance, where enrollment in plans with high inpatient coinsurance (>10%) was more likely for Black (1003 [30.1%]) and Hispanic (2302 [33.5%]) birthing people than Asian (1569 [18.3%]) or White (12 600 [18.4%]) birthing people (Table 3). There were no major differences in enrollment in high-deductible (>$1000) plans across racial and ethnic groups (3317 [38.7%] for Asian, 1232 [37.0%] for Black, 2350 [34.2%] for Hispanic, and 24 515 [35.8%] for White).

**Table 3.** Differences in Health Insurance Benefit Design by Birthing Person Race and Ethnicity^a^

Health insurance benefit design	Race and ethnicity group, No. (%) of birthing episodes			
	Asian (n = 8572)	Black (n = 3331)	Hispanic (n = 6872)	White (n = 68 478)
Deductible level				
$0	1903 (22.2)	756 (22.7)	1505 (21.9)	16 503 (24.1)
$1-$1000	3352 (39.1)	1342 (40.3)	3024 (44.0)	27 460 (40.1)
>$1000	3317 (38.7)	1232 (37.0)	2350 (34.2)	24 515 (35.8)
Inpatient coinsurance level				
0%	5169 (60.3)	1882 (56.5)	3711 (54.0)	46 223 (67.5)
1%-10%	1843 (21.5)	446 (13.4)	859 (12.5)	9724 (14.2)
>10%	1569 (18.3)	1003 (30.1)	2302 (33.5)	12 600 (18.4)

^a^ All differences across race and ethnicity groups within benefit design categories were statistically significant at *P* < .001. Owing to missing data, numbers may not sum to totals in column headings.

## Discussion

In this cross-sectional study of a commercially insured population, there were significant differences in out-of-pocket spending for maternity episodes by race and ethnicity. White birthing people incurred the lowest out-of-pocket spending, and Black birthing people had the highest out-of-pocket spending, both overall and as a percentage of the median household income in their census tracts. Although the level of out-of-pocket spending was higher during delivery than the prenatal period, the differences in out-of-pocket spending by race and ethnicity were larger during the prenatal period. Differences persisted after controlling for demographic and health risk factors that lead to higher maternity spending. Finally, though deductible payments comprised more than half of out-of-pocket spending, coinsurance was the chief contributor to racial differences in out-of-pocket spending, reflecting higher enrollment in health plans with high inpatient coinsurance exposure among Black and Hispanic birthing people.

Cost sharing is a tool for reducing overuse of health care (moral hazard, in economic terms) by holding patients directly responsible for some of the cost of their care. However, high out-of-pocket costs can lead to forgone or delayed health care services, even when they are high value, leading to worse health outcomes.^11,12^ Within the maternity episode, this is of particular concern in the prenatal period, when there is more patient discretion over the frequency and types of care received. We found that birthing people who were Black or Hispanic paid 74.4% and 51.2% more out-of-pocket, respectively, for recommended prenatal care services than birthing people who were White. This raises the concern that these differential costs could contribute to racial disparities in utilization and, ultimately, health outcomes.

Moreover, high out-of-pocket costs can create financial hardship, leading to challenges affording food, housing, and other essentials that can affect health outcomes.^30^ These differences in maternity episode out-of-pocket spending that we observe amount to a disproportionate share of median household income for Black and Hispanic birthing people. Unaffordable childbirth costs can lead to financial strain and medical debt.^13,14,15,16,17,18,19^ A recent study found that individuals with commercial insurance and lower incomes faced high financial strain from childbirth, with nearly half still owing money for childbirth costs 1 year later.^19^

Our results point to specific policy options that could address these concerns. Benefit design is an actionable area for policy change because decisions are driven by employers and payers, with state and federal policymakers establishing relevant guidelines. If stakeholders aim to reduce the out-of-pocket cost burden of maternity care, our findings suggest that lower deductibles would have the greatest contribution to reducing out-of-pocket costs for all groups. However, lower coinsurance would have the largest impact on reducing differences across racial and ethnic groups. Policy efforts to completely eliminate cost sharing for childbirth, such as Massachusetts’ proposed legislation, which BCBSMA has supported,^31^ would have the largest impact on reducing out-of-pocket spending and its differences across racial and ethnic groups.^32^ Persons with low income and employer-sponsored insurance could also enroll in Medicaid as secondary insurance if they qualify.^19^ Medicaid prohibits cost sharing for pregnancy-related services and would wrap around eligible individuals’ commercial insurance to cover out-of-pocket costs.

To continue to inform policy, future work should examine the implications of differences in out-of-pocket spending for racial and ethnic disparities in health care utilization and outcomes. More work is also needed to determine the drivers of these differences. In addition to benefit design and the health and demographic characteristics we control for in our analyses, the differences we observe may also reflect use of different providers (who negotiate different prices) or the amount and kind of care received. Care received can, in turn, be a result of different clinician behavior, patient preferences, and/or patients’ health status. Determining the drivers of these differences in spending is beyond the scope of this report.

### Limitations

This study has some limitations. First, this analysis used data from a single health insurance carrier and, although approximately one-quarter of the sample is from outside New England, it is not representative of all deliveries for birthing people with commercial health insurance. This sample had a slightly lower proportion of Black and higher proportion of White birthing people when compared with births in Massachusetts, New England, or nationwide (eTable 4 in Supplement 1). Second, our measure of race and ethnicity was imputed for most of the sample. The imputations had moderate to high sensitivity and specificity when compared with self-reported race and ethnicity, and prior studies have shown better performance with multiple imputations relative to complete case analyses, particularly when there is a large proportion of missing data.^33,34^ Third, we used census tract median income as a proxy for individual income, which we did not observe in the data. The individuals in our dataset, all of whom have commercial insurance, may not be representative of their census tract. Finally, we report descriptive analyses and results from regressions that are associations and should not be interpreted as causal. The intent of this analysis is to determine whether differences in out-of-pocket spending by race and ethnicity exist; further research should investigate the causes, consequences, and effects of interventions to reduce these differences.

## Conclusions

In this cross-sectional study of commercially insured birthing people during their maternity episodes, those who were Asian, Black, or Hispanic had higher out-of-pocket spending overall and as a proportion of median household income in their census block than White birthing people. Differences in benefit design, particularly coinsurance levels, were associated with these spending differences. These out-of-pocket spending differences are another way in which people of different races and ethnicities differentially experience maternity care in the US. Changes to benefit design could reduce out-of-pocket spending and differences across racial and ethnic groups. To the extent that exposure to high out-of-pocket costs can impact maternal health, these health insurance benefit design changes could offer a pathway to improving health equity.

## Notes

Supplement 1. **eTable 1.** Performance Characteristics of Imputation Models for Race and Ethnicity **eFigure.** STROBE Figure for Sample Construction **eTable 2.** *CPT*, *ICD-10*, and DRG Codes Used to Identify Deliveries **eTable 3.** Services Classified as Prenatal Services **eTable 4.** Race and Ethnicity of Commercially Insured Birthing People in Massachusetts, New England, and Nationwide, 2018 to 2022 **eTable 5.** Median Out-of-Pocket Spending by Race and Ethnicity for Total Episode, Delivery, Prenatal, and 6-Week Postpartum Periods **eTable 6.** Average Patient Out-of-Pocket Spending as Percentage of Census Tract Median Household Income **eTable 7.** Full Results for Regression of Total Maternity Out-of-Pocket Spending on Characteristics of the Birthing Person **eTable 8.** Maternity Episode Out-of-Pocket Spending Differences Between Race and Ethnicity Groups, Adjusted for Demographic and Health Status, with Clustered Standard Errors **eTable 9.** Maternity Episode Out-of-Pocket Spending Differences Between Race and Ethnicity Groups, Adjusted for Demographic and Health Status, as a Generalized Linear Model With a Gamma Distribution and Log Link **eTable 10.** Prenatal, Delivery, Postpartum, and Total Episode Out-of-Pocket Spending by Type of Cost Sharing

Supplement 2.
Data Sharing Statement
