# Supplementary material for: Racial and Ethnic Differences in Out-of-Pocket Spending for Maternity Care
Source: JAMA Health Forum. 2025 Feb 28;6(2):e245565. doi: 10.1001/jamahealthforum.2024.5565 (PMC11871542; doi:10.1001/jamahealthforum.2024.5565)
Supplement: Supplement 1. — eTable 1. Performance Characteristics of Imputation Models for Race and Ethnicity eFigure. STROBE Figure for Sample Construction eTable 2. CPT, ICD-10, and DRG Codes Used to Identify Deliveries eTable 3. Services Classified as Prenatal Services eTable 4. Race and Ethnicity of Commercially Insured Birthing People in Massachusetts, New England, and Nationwide, 2018 to 2022 eTable 5. Median Out-of-Pocket Spending by Race and Ethnicity for Total Episode, Delivery, Prenatal, and 6-Week Postpartum Periods eTable 6. Average Patient Out-of-Pocket Spending as Percentage of Census Tract Median Household Income eTable 7. Full Results for Regression of Total Maternity Out-of-Pocket Spending on Characteristics of the Birthing Person eTable 8. Maternity Episode Out-of-Pocket Spending Differences Between Race and Ethnicity Groups, Adjusted for Demographic and Health Status, with Clustered Standard Errors eTable 9. Maternity Episode Out-of-Pocket Spending Differences Between Race and Ethnicity Groups, Adjusted for Demographic and Health Status, as a Generalized Linear Model With a Gamma Distribution and Log Link eTable 10. Prenatal, Delivery, Postpartum, and Total Episode Out-of-Pocket Spending by Type of Cost Sharing [file jamahealthforum-e245565-s001.pdf]

Supplementary Online Content

Gourevitch RA, Cohen JL, Shakley T, et al. Racial and ethnic differences in out-of-pocket spending for maternity care. *JAMA Health Forum*. Published online February 28, 2025. doi:10.1001/jamahealthforum.2024.5565

eTable 1. Performance Characteristics of Imputation Models for Race and Ethnicity
eFigure. STROBE Figure for Sample Construction
eTable 2. *CPT*, *ICD-10*, and DRG Codes Used to Identify Deliveries
eTable 3. Services Classified as Prenatal Services
eTable 4. Race and Ethnicity of Commercially Insured Birthing People in Massachusetts, New England, and Nationwide, 2018 to 2022
eTable 5. Median Out-of-Pocket Spending by Race and Ethnicity for Total Episode, Delivery, Prenatal, and 6-Week Postpartum Periods
eTable 6. Average Patient Out-of-Pocket Spending as Percentage of Census Tract Median Household Income
eTable 7. Full Results for Regression of Total Maternity Out-of-Pocket Spending on Characteristics of the Birthing Person
eTable 8. Maternity Episode Out-of-Pocket Spending Differences Between Race and Ethnicity Groups, Adjusted for Demographic and Health Status, with Clustered Standard Errors
eTable 9. Maternity Episode Out-of-Pocket Spending Differences Between Race and Ethnicity Groups, Adjusted for Demographic and Health Status, as a Generalized Linear Model With a Gamma Distribution and Log Link
eTable 10. Prenatal, Delivery, Postpartum, and Total Episode Out-of-Pocket Spending by Type of Cost Sharing

This supplementary material has been provided by the authors to give readers additional information about their work.

© 2025 Gourevitch RA et al. *JAMA Health Forum*.

eTable 1. Performance Characteristics of Imputation Models for Race and Ethnicity

Race/Ethnicity	Positive Predictive Value (PPV)	Negative Predictive Value (NPV)	Sensitivity	Specificity
Asian	90.9%	99.5%	95.1%	99.1%
Black	83.1%	99.4%	81.2%	99.5%
Hispanic	87.7%	98.5%	74.8%	99.4%
White	95.3%	96.1%	99.1%	81.9%

Note: Birthing people with other races, including American Indian/Alaska Native, Native Hawaiian/Other Pacific Islander, Other, and Multiracial are excluded due to a lack of sufficient self-reported data to assess the accuracy of imputed data for these categories.

© 2025 Gourevitch RA et al. *JAMA Health Forum*.

eFigure. STROBE Figure for Sample Construction

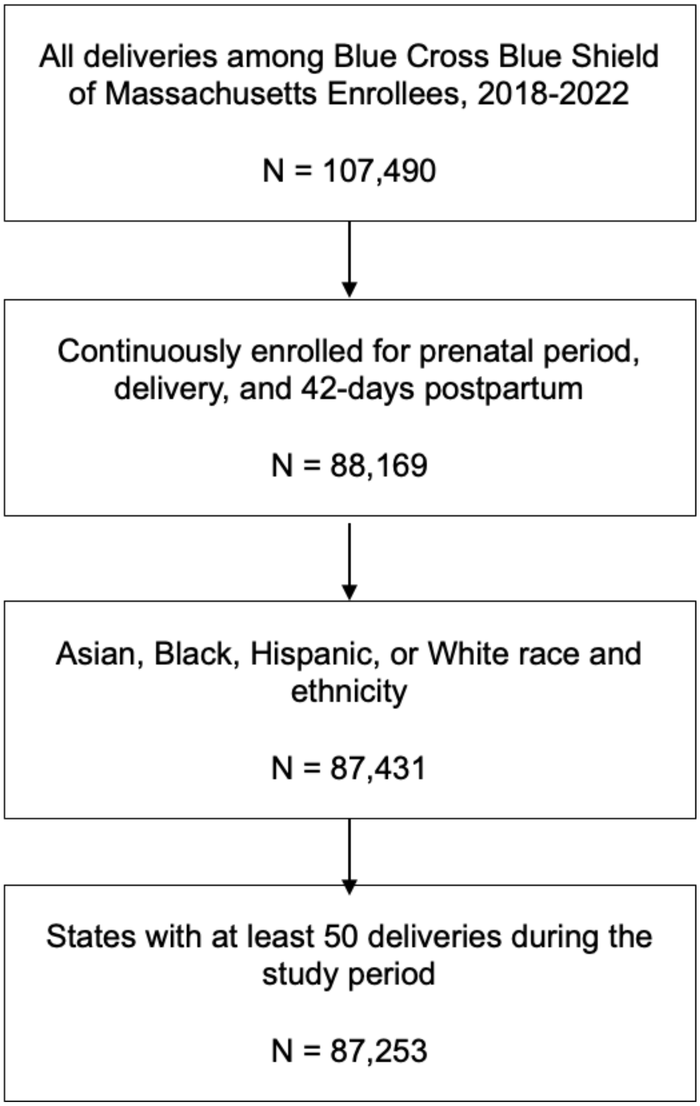

© 2025 Gourevitch RA et al. *JAMA Health Forum*.

eTable 2. *CPT*, *ICD-10*, and DRG Codes Used to Identify Deliveries

Code Type	Code	Cesarean Section	Code Description
CPT	59510	X	Routine obstetric care including antepartum care, cesarean delivery, and postpartum care
CPT	59514	X	Cesarean delivery only
CPT	59515	X	Cesarean delivery only; including postpartum care
CPT	59618	X	Routine obstetric care including antepartum care, cesarean delivery, and postpartum care, following attempted vaginal delivery after previous cesarean delivery
CPT	59620	X	Cesarean delivery only, following attempted vaginal delivery after previous cesarean delivery
CPT	59622	X	Cesarean delivery only, following attempted vaginal delivery after previous cesarean delivery; including postpartum care
CPT	59400		Routine obstetric care including antepartum care, vaginal delivery (with or without episiotomy, and/or forceps) and postpartum care
CPT	59409		Vaginal delivery only (with or without episiotomy and/or forceps);
CPT	59410		Vaginal delivery only (with or without episiotomy and/or forceps); including postpartum care
CPT	59610		Routine obstetric care including antepartum care, vaginal delivery (with or without episiotomy, and/or forceps) and postpartum care, after previous cesarean delivery
CPT	59612		Vaginal delivery only, after previous cesarean delivery (with or without episiotomy and/or forceps)
CPT	59614		Vaginal delivery only, after previous cesarean delivery (with or without episiotomy and/or forceps); including postpartum care
DRG	540	X	Cesarean Delivery
DRG	541		Vaginal Delivery W Sterilization &/Or D&C
DRG	541		Vaginal Delivery W/ Sterilization &/Or D&C
DRG	542		Vaginal Delivery W/ Complicating Procedures Exc Sterilization &/Or D&C
DRG	542		Vaginal Delivery W/ Complicating Procedures Exc St
DRG	560		Vaginal Delivery
DRG	766	X	Cesarean Section Without Cc/Mcc
DRG	768		Vaginal Delivery W O.R. Proc Except Steril &/Or D&C
DRG	774		Vaginal Delivery With Complicating Diagnoses
DRG	775		Vaginal Delivery W/O Complicating Diagnoses
DRG	775		Vaginal Delivery Without Complicating Diagnoses
ICD10 Proc	10D00Z0		Extraction of Products of Conception High Open Approach
ICD10 Proc	10D00Z1		Extraction of Products of Conception Low Open Approach
ICD10 Proc	10D00Z2		Extraction of Products of Conception Extraperitoneal Open Approach
ICD10 Proc	10D07Z3		Extraction of Products of Conception Low Forceps Via Opening
ICD10 Proc	10D07Z4		Extraction of Products of Conception Mid Forceps Via Opening
ICD10 Proc	10D07Z5		Extraction of Products of Conception High Forceps Via Opening
ICD10 Proc	10D07Z6		Extraction of Products of Conception Vacuum Via Opening
ICD10 Proc	10D07Z7		Extraction of Products of Conception Int Version Via Opening
ICD10 Proc	10D07Z8		Extraction of Products of Conception Other Via Opening
ICD10 Proc	10E0XZZ		Delivery of Products of Conception External Approach

eTable 3. Services Classified as Prenatal Services

Category	Codes used to identify services in claims data
STI Tests	Z114*, 87806, 87534, 87535, 87536, 87537, 87538, 87539, 87390, 87391, 87389, 3292F, 3490F, 3491F, 3492F, 3494F, 3496F, 3497F, 3498F, 3500F, 3502F, 3503F, G0432, G0433, G0435, S3645, G0475, 86689, 86701, 86702, 86703, 80081, 86592, 86593, 3512F, 0065U, 87285, G9228, 86781, 86780, 0064U, 80055, 80074 ,86704, 86705, 86706, 87340, 87341, 87516, 87517, G8869, G9912
Obstetrical Lab Panels	86901, 86906, 3290F, 3291F, 3293F, 80055, 80081, 86850, 86860, 86870, 86905, 86976, 86975, 86977, 86971, 86970, 86972, 86978, 86762, 86765, 85004, 85007, 85009, 85013, 85014, 85018, 85025, 85027, 85032, 85041, 85044, 85045, 85046, 85048, 85049, G0306, G0307, 80050
Ultrasounds & Anatomy Scans	76805, 76810, 76811, 76812, 76815, 76816, 76817, 76801, 76802, 76813, 76814
Oral Glucose Tolerance Test	82950, 82951, 82947
TDAP Vaccine	90696, 90697, 90698, 90700, 90701, 90714, 90715, 90471, 90472, 90460, 90461
Group B Streptococcus	Z3685*, 87150, 3294F, 87802, 87653, 87801, 87081, 87084, 87070, 87077, 87147
Fetal Testing	59025, 76818, 76819, 76820, 76821
Genetic Testing	81200, 81205, 81209, 81220, 81242, 81250, 81251, 81255, 81257, 81260, 81290, 81329, 81330, 81361, 81412, 81443, S3835, S3847, S3848, S3849, S3850, S3851, 81508, 81509, 81510, 81511, 81512, 0124U, 0125U, 0126U, 82105, 82106, 82677, 84163, 86336, 81420, 81422, 81507, 0009M, 0168U, 81228, 81229, S3870

Note: All codes are CPT codes except those marked with *, which are ICD-10 diagnosis codes. Other services during the prenatal period with the clinician type listed as OB/GYN were also included in the definition of prenatal services.

© 2025 Gourevitch RA et al. *JAMA Health Forum*.

eTable 4. Race and Ethnicity of Commercially Insured Birthing People in Massachusetts, New England, and Nationwide, 2018 to 2022

	Analytic Sample	Massachusetts	New England	United States
Asian	9.8%	10.7%	8.3%	8.9%
Black	3.8%	6.3%	5.6%	8.8%
Hispanic	7.9%	11.8%	10.2%	15.3%
White	78.5%	71.2%	76.0%	67.0%

Notes: Data on the racial and ethnic composition of commercially insured births from 2018-2022 in Massachusetts, New England, and the United States come from CDC Wonder's Natality database. The New England states correspond to Health and Human Services (HHS) Region 1 (Connecticut, Maine, Massachusetts, New Hampshire, Rhode Island, and Vermont).

© 2025 Gourevitch RA et al. *JAMA Health Forum*.

eTable 5. Median Out-of-Pocket Spending by Race and Ethnicity for Total Episode, Delivery, Prenatal, and 6-Week Postpartum Periods

	Median Out-of-Pocket Spending			
**Race and Ethnicity**	**Total Episode**	**Delivery**	**Prenatal Period**	**Postpartum Period**
Asian	$1,846.53	$1,253.25	$164.26	$0.00
Black	$2,099.83	$1,127.61	$276.38	$0.00
Hispanic	$2,058.99	$1,201.60	$262.48	$0.00
White	$1,597.92	$976.05	$214.47	$0.00

Notes: Spending inflated to 2022 dollars using the Medical Consumer Price Index

© 2025 Gourevitch RA et al. *JAMA Health Forum*.

eTable 6. Average Patient Out-of-Pocket Spending as Percentage of Census Tract Median Household Income

Race and Ethnicity	10th percentile	25th percentile	Median	Mean	75th percentile	90th percentile
**Prenatal Period**						
Asian	0.0%	0.0%	0.2%	0.6%	0.6%	1.5%
Black	0.0%	0.1%	0.4%	1.2%	1.2%	3.0%
Hispanic	0.0%	0.1%	0.3%	0.9%	1.0%	2.4%
White	0.0%	0.0%	0.2%	0.6%	0.6%	1.5%
**Delivery Period**						
Asian	0.0%	0.3%	1.2%	2.0%	2.8%	4.9%
Black	0.0%	0.2%	1.5%	2.8%	3.9%	6.9%
Hispanic	0.0%	0.3%	1.5%	2.6%	3.7%	6.5%
White	0.0%	0.2%	0.9%	1.7%	2.4%	4.3%
**Postpartum Period**						
Asian	0.0%	0.0%	0.0%	0.1%	0.0%	0.2%
Black	0.0%	0.0%	0.0%	0.1%	0.1%	0.3%
Hispanic	0.0%	0.0%	0.0%	0.1%	0.1%	0.3%
White	0.0%	0.0%	0.0%	0.1%	0.1%	0.2%
**Total Episode**						
Asian	0.1%	0.6%	1.8%	2.6%	3.5%	6.0%
Black	0.2%	0.9%	2.6%	4.1%	5.5%	9.4%
Hispanic	0.3%	0.9%	2.4%	3.6%	5.0%	8.3%
White	0.2%	0.6%	1.5%	2.4%	3.2%	5.5%

Notes: Median Household income per delivery is measured for the census block group where the birthing person lived during the maternity episode. The total maternity episode includes the prenatal period, delivery, and 42 days postpartum. Means are statistically significantly different across race and ethnicity groups for each spending period (p<0.001). Regression analyses used multiple imputation to account for imputed data. Spending inflated to 2022 dollars using the Medical Consumer Price Index.

© 2025 Gourevitch RA et al. *JAMA Health Forum*.

eTable 7. Full Results for Regression of Total Maternity Out-of-Pocket Spending on Characteristics of the Birthing Person

	Coefficient	95% Confidence Interval
Race and Ethnicity		
Asian	$122.34	($82.34, $162.35)
Black	$251.88	($190.03, $313.72)
Hispanic	$91.95	($47.22, $136.68)
White	Ref.	
Maternal Age Group (Years)		
0-19	$190.12	($55.76, $324.49)
20-24	$142.25	($89.03, $195.47)
25-29	$62.37	($28.83, $95.91)
30-34	Ref.	
35-39	-$43.26	($-75.09, $-11.44)
40-44	$18.40	($-37.8, $74.59)
>=45	-$39.01	($-227.93, $149.92)
Mode of Delivery		
Vaginal	Ref.	
Cesarean	$77.52	($51.3, $103.74)
Leonard Comorbidity Index		
Quartile 1 (Lowest Risk)	Ref.	
Quartile 2	$48.56	($10.93, $86.18)
Quartile 3	$51.20	($16.99, $85.41)
Quartile 4 (Highest Risk)	$110.50	($74.6, $146.39)
Length of Stay for Delivery		
Tertile 1 (Shortest Stay)	Ref.	
Tertile 2	$31.60	($-8.27, $71.48)
Tertile 3 (Longest Stay)	$96.18	($56.39, $135.96)
Intercept	$1,215.89	($1111.64, $1320.13)

Notes: Model also includes fixed effects for each month-year of delivery and for the birthing person's state of residence. Length of stay tertiles are calculated within the birthing person's delivery mode group.

eTable 8. Maternity Episode Out-of-Pocket Spending Differences Between Race and Ethnicity Groups, Adjusted for Demographic and Health Status, with Clustered Standard Errors

	**Coefficient**	**95% Confidence Interval**
Race and Ethnicity		
Asian	$122.34	($79.78, $164.91)
Black	$251.88	($180.1, $323.65)
Hispanic	$91.95	($44.3, $139.6)
White	Ref.	
Maternal Age Group (Years)		
0-19	$190.12	($47.07, $333.18)
20-24	$142.25	($83.84, $200.65)
25-29	$62.37	($27.51, $97.23)
30-34	Ref.	
35-39	-$43.26	($-75.33, $-11.19)
40-44	$18.40	($-39.74, $76.54)
>=45	-$39.01	($-232.79, $154.77)
Mode of Delivery		
Vaginal	Ref.	
Cesarean	$77.52	($49.88, $105.17)
Leonard Comorbidity Index		
Quartile 1 (Lowest Risk)	Ref.	
Quartile 2	$48.56	($10.79, $86.32)
Quartile 3	$51.20	($16.73, $85.67)
Quartile 4 (Highest Risk)	$110.50	($73.58, $147.42)
Length of Stay for Delivery		
Tertile 1 (Shortest Stay)	Ref.	
Tertile 2	$31.60	($-8.98, $72.19)
Tertile 3 (Longest Stay)	$96.18	($55.45, $136.91)
Intercept	$1,215.89	($1115.84, $1315.94)

Notes: Model also includes fixed effects for each month-year of delivery and for the birthing person’s state of residence. Length of stay tertiles are calculated within the birthing person’s delivery mode group. Standard errors are clustered at the member level.

© 2025 Gourevitch RA et al. *JAMA Health Forum*.

eTable 9. Maternity Episode Out-of-Pocket Spending Differences Between Race and Ethnicity Groups, Adjusted for Demographic and Health Status, as a Generalized Linear Model With a Gamma Distribution and Log Link

	Coefficient	95% Confidence Interval
Race and Ethnicity		
Asian	0.06	(0.04, 0.09)
Black	0.10	(0.06, 0.13)
Hispanic	0.04	(0.02, 0.07)
White	Ref.	
Maternal Age Group (Years)		
0-19	0.07	(-0.005, 0.15)
20-24	0.04	(0.009, 0.07)
25-29	0.02	(0.001, 0.04)
30-34	Ref.	
35-39	-0.02	(-0.03, 0.003)
40-44	0.01	(-0.02, 0.04)
>=45	0.01	(-0.1, 0.12)
Mode of Delivery		
Vaginal	Ref.	
Cesarean	0.03	(0.02, 0.05)
Leonard Comorbidity Index		
Quartile 1 (Lowest Risk)	Ref.	
Quartile 2	0.02	(0.001, 0.05)
Quartile 3	0.02	(-0.001, 0.04)
Quartile 4 (Highest Risk)	0.04	(0.02, 0.06)
Length of Stay for Delivery		
Tertile 1 (Shortest Stay)	Ref.	
Tertile 2	0.02	(-0.004, 0.04)
Tertile 3 (Longest Stay)	0.06	(0.03, 0.08)
Intercept	7.18	(7.12, 7.24)

Notes: Model also includes fixed effects for each month-year of delivery and for the birthing person’s state of residence. Length of stay tertiles are calculated within the birthing person’s delivery mode group

© 2025 Gourevitch RA et al. *JAMA Health Forum*.

eTable 10. Prenatal, Delivery, Postpartum, and Total Episode Out-of-Pocket Spending by Type of Cost Sharing

Race	Deductible	Copayments	Coinsurance
		Prenatal Period	
Asian	$348.53	$73.09	$49.45
Black	$446.78	$133.68	$84.42
Hispanic	$375.41	$132.66	$78.84
White	$333.05	$124.48	$44.69
		Delivery Period	
Asian	$954.73	$102.36	$606.74
Black	$856.60	$116.91	$669.35
Hispanic	$826.85	$115.40	$685.75
White	$845.16	$149.08	$453.81
		Postpartum Period	
Asian	$30.00	$23.62	$12.57
Black	$36.22	$32.99	$18.04
Hispanic	$35.51	$32.74	$14.90
White	$32.27	$39.81	$12.06
		Total Episode	
Asian	$1,333.27	$199.07	$668.76
Black	$1,339.59	$283.59	$771.81
Hispanic	$1,237.76	$280.80	$779.49
White	$1,210.47	$313.38	$510.56

Notes: Spending inflated to 2022 dollars using the Medial CPI. The Postpartum Period includes 42 days following delivery discharge. The total maternity episode includes the prenatal period, delivery, and 42 days postpartum.

© 2025 Gourevitch RA et al. *JAMA Health Forum*.
