# Supplementary material for: Racial and Ethnic Differences in Out-of-Pocket Spending for Maternity Care
Source: JAMA Health Forum. 2025 Feb 28;6(2):e245565. doi: 10.1001/jamahealthforum.2024.5565 (PMC11871542; doi:10.1001/jamahealthforum.2024.5565)
Supplement: Supplement 2. — Data Sharing Statement [file jamahealthforum-e245565-s002.pdf]

Data Sharing Statement

Gourevitch. Racial and Ethnic Differences in Out-of-Pocket Spending for Maternity Care. *JAMA Health Forum*. Published February 28, 2025. doi:10.1001/jamahealthforum.2024.5565

Data
Data available: No

Additional Information
Explanation for why data not available: Our study uses identifiable health insurance enrollment and claims data and it is not permissible to make these data publicly available.
